# Predictors of posttraumatic stress and appetitive aggression in active soldiers and former combatants

**DOI:** 10.3402/ejpt.v6.26553

**Published:** 2015-04-21

**Authors:** Corina Nandi, Anselm Crombach, Manassé Bambonye, Thomas Elbert, Roland Weierstall

**Affiliations:** 1 Department of Psychology, University of Konstanz, Konstanz, Germany; 2 Department of Psychology, University Lumière of Bujumbura, Bujumbura, Burundi

**Keywords:** PTSD, trauma, aggression, childhood maltreatment, combatants, violence, Burundi, risk factors

## Abstract

**Background:**

During the period between 1993 and 2005, the people of Burundi were trapped within a violent civil war. In post-conflict regions, symptoms of posttraumatic stress disorder (PTSD) were found to be widespread. At the same time, combatants often reported having perceived committing violence as exciting and appealing, an experience referred to as appetitive aggression. Both of these phenomena hamper the building of a functional and peaceful society.

**Objective:**

This study aims to investigate the factors that are associated with the level of PTSD and appetitive aggression in former and still active combatants.

**Methods:**

Semi-structured interviews were conducted with 948 male Burundians: 556 active soldiers and 392 ex-combatants. PTSD symptom severity was assessed using the PTSD Symptom Scale Interview, while appetitive aggression was assessed using the Appetitive Aggression Scale.

**Results:**

Linear regression analyses revealed that the number of traumatic events, childhood maltreatment, and their interaction predicted PTSD symptom severity, whereas self-committed violence did not. The number of traumatic events and self-committed violence were associated with appetitive aggression. Childhood maltreatment alone was not associated with appetitive aggression; however, its interaction with self-committed violence did predict appetitive aggression. When controlling for predictors, ex-combatants reported a higher degree of PTSD symptomatology, whereas active soldiers reported a higher degree of appetitive aggression.

**Conclusion:**

We conclude that childhood maltreatment is an additional, significant risk factor that exacerbates the psychological consequences of violent conflicts. Self-committed violence may not necessarily engender trauma-related disorders, but is highly related to appetitive aggression.

Since 1965, Burundi has been the scene of violent conflicts, with violence breaking out at least once every decade. The longest and most recent episode of conflict lasted from 1993 until 2005 (Watt, 2008). It is estimated that more than a quarter of a million people lost their lives and that over a million people from a population of 10 million were displaced as a consequence of the civil war (Uvin, 2009). The war was characterized by violence and atrocities against the civilian population as Tutsi-dominated national army and Hutu rebel groups fought for control. Following the peace agreement in 2005, demobilization of both the army and rebel groups began. Some members of the rebel groups were integrated into the army to form the national Burundian army that is still active. No novice combatants have been recruited for the army since the end of the civil war. As a consequence, both former and currently active combatants in Burundi represent a population that has experienced numerous and diverse war events.

Numerous studies of war-affected populations have demonstrated that repeated exposure to different types of traumatic events cumulatively increases the risk of PTSD. This relationship is also known as the “building block effect” (Kolassa et al., 2010; Neuner et al., 2004). Throughout Burundi, symptoms of PTSD were found withinthe general population (Yeomans, Forman, Herbert, & Yuen, 2010). However, researchers have yet to investigate the existence of PTSD symptomology in a population of Burundian combatants specifically. Studies with comparable populations of combatants in other conflict regions in East-Africa found that such individuals suffered from substantial mental health impairment and, in particular, PTSD symptoms (Bayer, Klasen, & Adam, 2007; Hecker, Hermenau, Maedl, Schauer, & Elbert, 2013; Pfeiffer & Elbert, 2011). Experiencing war as a combatant includes the exposure to horrifying events such as injury or witnessing the death of comrades or civilians. Especially in civil wars, combatants additionally take on the role of perpetrating violent acts. Several studies have postulated that the perpetration of violent acts is a potentially traumatic experience that enhances the risk of developing PTSD (Bayer et al., 2007; Maguen et al., 2010; Van Winkle & Safer, 2011). However, in a study with combatants in the Democratic Republic of the Congo, perpetrating violence was found to be traumatic for forcibly recruited combatants, but not for combatants who volunteered themselves (Hecker et al., 2013).

In the context of war and combat, PTSD symptoms are often viewed as direct consequences of traumatic war experiences. By now, however, there are a large number of studies showing that other factors play an important role in the development and maintenance of PTSD symptoms in individuals exposed to war (Brewin, Andrews, & Valentine, 2000; Dohrenwend, Yager, Wall, & Adams, 2013; Fontana & Rosenheck, 1994; King, King, & Foy, 1996). In particular, childhood maltreatment was found to substantially increase the risk of PTSD following exposure to subsequent traumatic events (Catani et al., 2010; Iversen et al., 2007; Van Voorhees et al., 2012). The effect of childhood adversities, such as physical, sexual, or verbal abuse, and also neglect in the development of PTSD could also be demonstrated in recent prospective studies of western soldiers deployed in foreign missions (Berntsen et al., 2012; Polusny et al., 2011). In the context of the Burundian civil war, the situation for the combatants was different in comparison to soldiers participating in foreign missions with peaceful conditions in their home country. Burundian combatants were both victims and perpetrators of the civil war, and a portion of the combatants fighting in this war were still children when the war started. Research demonstrated that childhood maltreatment is more common in families that are affected by war (Catani, Jacob, Schauer, Kohila, & Neuner, 2008). Thus, maltreatment experienced by caregivers as children and traumatic war experiences are not completely independent of one another. Still, or precisely because of that, it is important to clarify the individual contributions of these diverse types of experiences to the development of PTSD symptoms in the context of war.

In addition to PTSD symptomatology, the experience of war and violence is often also associated with increased violent and aggressive behavior in individuals (Schauer & Elbert, 2010). Research has repeatedly demonstrated that PTSD symptoms (and in particular hyperarousal) may trigger violent behavior caused by anger and hostility (Elbogen, Wagner, Calhoun, Fuller, & Kinneer, 2010; Jakupcak et al., 2007). This affective and impulsive type of aggressive behavior often occurs in reaction to a perceived threat, and is referred to as reactive aggression (Anderson & Bushman, 2002; Fontaine, 2007; Hubbard et al., 2002; Vitiello & Stoff, 1997). Instrumental or proactive aggression, on the other hand, is planned, purposeful, and goal-oriented. Such aggression is adopted in order to achieve some positive outcome, driven therefore by a secondary reward such as social status (Fontaine, 2006; Schwartz et al., 1998). However, reports of combatants and individuals perpetrating violence have indicated that committing violent acts can also be perceived as fascinating, appealing and exciting (e.g., Crombach, Weierstall, Hecker, Schalinski, & Elbert, 2013; Elbert, Weierstall, & Schauer, 2010). In addition to aggression driven by perceived threat or secondary reward, there is another type of aggression evoked by the rewarding cues of the aggressive act itself. The perpetration of violence or the infliction of harm on a victim occurs for the purpose of satisfying violence-related passion. This kind of aggression is referred to as appetitive aggression (Elbert et al., 2010; Weierstall & Elbert, 2011). We investigated appetitive aggression in different populations of ex-combatants. In these studies, a considerable proportion of participants reported perceiving violence and aggression as positive and thrilling (e.g., Hecker et al., 2013; Weierstall, Schaal, Schalinski, Dusingizemungu, & Elbert, 2011). The high prevalence of appetitive aggression in these studies indicates that this is not limited to a psychopathological subgroup, but is rather a common trait in the context of war. Combatants and soldiers involved in armed conflict have to inflict violence as part of their job and duty. Committing violence may be necessary to survive in a violent environment. Hence, appetitive aggression facilitating violent or cruel behavior can have adaptive and advantageous functions in such a context.

While potential predictors for PTSD symptoms are well defined in literature, the factors fostering appetitive aggression have only recently begun to be investigated. Reports from former combatants depict a gradual shift in their perception of killing and atrocities: in the beginning these acts were frightening, but after a time they became used to it and even found it exciting (Maclure & Denov, 2006). As this gradual transformation in the perception of violence implies, several studies have also demonstrated that the perpetration of aggressive acts predicts appetitive aggression (Crombach et al., 2013; Hecker et al., 2012; Weierstall et al., 2011). Since appetitive aggression can be seen as an adaptive mechanism when living in a cruel environment, not only perpetrating violence but also witnessing cruelty or experiencing it by oneself could be positively associated with appetitive aggression. Prior studies revealed ambivalent findings concerning the relationship between traumatic experiences and appetitive aggression (Hecker et al., 2012; Weierstall et al., 2011). To date, the role of childhood maltreatment in influencing the level of appetitive aggression experienced by an individual has not been investigated. However, it should be considered as a potential predictor, as insecure and violent environments seem to facilitate the development of violent behavior, promoting a cycle of violence (Crombach & Elbert, 2014; Elbert, Rockstroh, Kolassa, Schauer, & Neuner, 2006).

Both PTSD symptoms and appetitive aggression are widespread amongst populations of combatants (Hecker et al., 2013; Weierstall et al., 2011). Which types of experiences predict these two phenomena so prevalent in conflict settings? Do the same factors underlie both or do different experiences play a role in their emergence? The present study aims to disentangle the influential factors of PTSD symptoms and appetitive aggression in a sample of male Burundian combatants. A portion of the combatants had been demobilized after the end of the civil war whereas, the other segment remains actively enlisted in the Burundian military. As the demobilization process might have influenced the present mental health state of the combatants and former combatants, we examined differences between these two groups.

We hypothesized that the experience of traumatic events would be the best predictor for current PTSD symptom severity, whereas, self-committed violent acts would best explain the variability of appetitive aggression. Childhood maltreatment was expected to be an important factor for PTSD symptomatology specifically, but we expected that it might also promote appetitive aggression in the sense of a circle of violence.

## Method

### Participants

We investigated 948 male Burundian combatants of which 392 had been demobilized after war and 556 were still active as soldiers. Those still active in the military were preparing for deployment in the African Union Mission for Somalia (AMISOM) at the time of our investigation. Participants in the demobilized group were contacted through an official national veteran association. Active soldiers were randomly chosen from two battalions of the Burundian army. Descriptives of both groups are shown in Table 1. The data for two active soldiers and one ex-combatant were excluded from analyses due to missing data in the main outcome variables.

**Table 1:** Descriptives of active soldiers and ex-combatants

Variable	Active soldiers (*n*=554)	Ex-combatants (*n*=391)	Statistics
Former rebel, No. (%)	184 (33.21)	129 (32.99)	*χ* ^*2*^ (1, 945) = 0.01
Traumatic event types, *M* (*SD*) [*range*]	10.02 (3.10) [0–17]	13.64 (2.39) [6–19]	*t*_935.92_=20.32***, *d*=1.28
Childhood maltreatment, *M* (*SD*) [*range*]	0.48 (0.82) [0–4]	1.06 (1.08) [0–4]	*t*_693.10_=8.97***, *d*=0.62
Self-committed violence, *M* (*SD*) [*range*]	4.04 (3.18) [0–13]	8.59 (3.23) [0–14]	*t*_943.00_=21.57***, *d*=1.42
PSS-I sum score, *M* (*SD*) [*range*]	3.04 (4.66) [0–32]	13.63 (11.31) [0–42]	*t*_484.10_=17.50***, *d*=1.31
AAS sum score, *M* (*SD*) [*range*]	19.75 (10.62) [0–46]	28.37 (14.51) [0–58]	*t*_672.84_=10.01***, *d =*0.70

*M*= mean, *SD*=standard derivation; asterisks indicate statistical significance: *p<0.05, **p<0.01

*** *p*<0.001.

Participation in the study was voluntary and all participants had to sign an informed consent sheet prior to the interview. In case of illiteracy, oral informed consent was collected. All participants agreed to take part in the research project. The ethics committees of the University of Konstanz, Germany, and of the University Lumière of Bujumbura, Burundi, approved the study. The active soldiers received no payment for their participation. A financial compensation equivalent to 5€ was paid to cover transportation costs for the ex-combatants. With respect to the special vulnerability of the population, anonymity and confidentiality were ensured through the electronic coding and storage of the data, which fulfilled the highest and most secure data encryption standards.

## Measures

### Traumatic event types

Exposure to different types of traumatic events was assessed using a checklist of 19 potentially traumatic war-related and non-war-related events (e.g., assault by weapon, and life-threatening accidents), which also included events from the checklist of the Posttraumatic Stress Diagnostic Scale (Foa, Cashman, Jaycox, & Perry, 1997). The checklist was a version of a previously published checklist (Neuner et al., 2004) that was adapted to the Burundian cultural context. It showed a high test–retest reliability (*r*=0.73, *p*<0.001) and significant accordance with the Composite International Diagnostic Interview (CIDI) Event List (Ertl et al., 2010) in an earlier study of former child soldiers in the Great Lakes Region of Africa. The exact frequency of a specific traumatic event was not measured, as this is considered to be unreliable due to memory biases (Kolassa et al., 2010). Instead, the items were coded dichotomously. If a participant had ever experienced an event, this was coded as “1,” otherwise it was coded as “0.” As a measurement of trauma load, the number of experienced traumatic event types was summed.

### Childhood maltreatment

Exposure to different types of childhood maltreatment was assessed by means of four single items oriented on the common domains of childhood maltreatment (physical abuse, verbal abuse, neglect and sexual abuse, cp. e.g., Teicher, Samson, Polcari, & McGreenery, 2006). The items were coded dichotomously, as “1” if the participant had experienced a type of childhood maltreatment and as “0” if not. As a measurement of childhood maltreatment, the number of experienced types of childhood maltreatment was summed. The following questions were asked: “Have your parents/caretaker neglected you during childhood?”, “Have your parents/caretaker regularly humiliated you verbally during childhood?”, “Have you been physically abused by your parents/caretaker during childhood?”, “Have you experienced a sexual assault by a family member/caretaker/friend or foreign person during childhood?”

### Self-committed violence

To measure self-committed violence, we systematically assessed 14 different types of perpetrated violence (e.g., mutilation, rape or killing). The items were coded dichotomously and were summed up to create a self-committed violence sum score.

### PTSD symptom severity

Symptoms of PTSD were investigated using the PTSD Symptom Scale Interview (PSS-I) (Foa, Riggs, Dancu, & Rothbaum, 1993). The PSS-I is a semi-structured interview which consists of 17 items and has proven its validity in comparable East-African samples (Ertl et al., 2010). The items correspond to the 17 symptoms of PTSD in the DSM-IV, divided into the three clusters of re-experiencing, avoidance and hyperarousal. The assessment of symptom severity refers to the last 2 weeks and is based on a four-point Likert scale ranging from 0 (not at all) to 3 (five or more times per week/almost always). For analysis, a sum score of all symptoms was computed to assess PTSD symptom severity, with a possible range of 0–51. The PSS-I comes with good psychometric properties, with an internal consistency of Cronbach's α=0.86 (Foa & Tolin, 2000). In the present study, Cronbach's α was 0.94.

### Appetitive aggression

To assess experiences of appetitive violence we used the Appetitive Aggression Scale (AAS), a semi-structured interview that has been used and validated in other comparable populations with more than 1,600 participants (Weierstall & Elbert, 2011). The AAS consists of 15 items regarding the perception of violence or appetitive aggression (e.g., “Is it exciting for you if you make an opponent really suffer?” or “Once fighting has started, do you get carried away by the violence?”). The interviewer rated the level of the interviewee's agreement on a five-point Likert scale ranging from 0 (“I totally disagree”) to 4 (“I totally agree”). For analysis, a sum score with a possible range of 0–60 was computed. In the validation study, the AAS score showed a Cronbach's α coefficient of 0.85 and further analysis revealed that the AAS measures a distinct construct of human aggression (Weierstall & Elbert, 2011). In the present study, Cronbach's α was 0.87.

### Procedure

Interviews with the demobilized combatants were conducted at the campus of the Université Lumière in Bujumbura, Burundi. Interviews with the active soldiers were mostly conducted at the military camp Mudubugu (Bubanza province, Burundi), and a smaller number were conducted at other military camps in Gakumbu (Bujumbura rural, Burundi) and Bubanza province (Burundi). At the time of the study, the active soldiers were receiving a 2-month training session in preparation for AMISOM. The training took place in these camps, with one battalion at a time. Six clinical psychologists of the University of Konstanz, four military psychologists of the Burundian army and six advanced students of clinical psychology from the University Lumière interviewed the participants. Interviews were conducted in Kirundi. Non-local interviewers conducted the interviews with the help of six bilingual interpreters. Different interpreters translated all questionnaires into Kirundi and back into English. The translations were discussed in detail with the interpreters before their application in the interview. All interviewers and interpreters had been trained in the concepts of mental disorders and aggression in an intensive 6-week training session prior to data collection and also received permanent supervision to ensure data quality. The interviews lasted one and a half hours on average.

### Data analysis

Robust multiple linear regression analyses were conducted to investigate the prediction of *PTSD symptom severity* as well as *appetitive aggression* by the number of *traumatic event types*, *childhood maltreatment* and *self-committed violence*. The aforementioned predictors as well as all two-way interactions were included in the regression models. To identify potential differences between ex-combatants and active soldiers, group assignment was included in the model and dummy-coded using 0 for the ex-combatants and 1 for the active soldiers. The predictors were mean-centered to reduce potential multicollinearity (Kleinbaum, Kupper, Nizam, & Muller, 2008). In a first step, all variables were simultaneously entered into the model. In a second step, the insignificant variables were excluded in a stepwise manner. The final model was selected according to Bayesian Information Criterion (BIC, Schwarz, 1978). All analyses used a two-tailed α=0.05.

## Results

### Predictors of PTSD symptom severity

We conducted a robust multiple linear regression to identify significant predictors of *PTSD symptom severity* and to assess if their influence differed between the ex-combatants and active soldiers. The results are presented in Table 2 (Model 1). The selected model revealed main effects for the group variable and the variables *traumatic event types* and *childhood maltreatment. Self-committed violence* was no additional predictor. Of all included interactions, the interaction of traumatic event types * childhood maltreatment significantly predicted *PTSD symptom severity*. Additionally, the interactions of *group* * *traumatic event types* and *group* * *childhood maltreatment* were significant, indicating a stronger impact of *traumatic event types* as well as childhood maltreatment on *PTSD symptom severity* in the group of the ex-combatants. The model accounted for 43% of the variance of *PTSD symptom severity* (*F* (6, 944) = 104.12, *p*<0.001, *f* ^2^=0.75). The maximum Variance Inflammation Factor did not exceed 1.84. Hence, multicollinearity could be discounted.

**Table 2:** Robust multiple linear regression analysis predicting PTSD symptom severity (Model 1) and appetitive aggression (Model 2)

	Model 1		Model 2	
	
	PTSD symptom severity		Appetitive aggression	
	
Predictor variables	*β*	*t*	*β*	*t*
Group^a^	−0.25	−10.41***	0.13	3.61***
Traumatic event types	0.29	11.93***	0.22	5.75***
Childhood maltreatment	0.18	7.45***	<−0.01	−0.07
Self-committed violence	–	–	0.52	14.07***
Traumatic event types * childhood maltreatment	0.11	4.18***	–	–
Traumatic event types * self-committed violence	–	–	–	–
Childhood maltreatment * self-committed violence	–	–	0.09	3.20***
Group^a^ * traumatic event types	−0.17	−6.48***	−0.10	−2.57*
Group^a^ * childhood maltreatment	−0.05	−2.39*	–	–
Group^a^ * self-committed violence	–	–	−0.09	−2.27*

*β*=standardized regression coefficient. *R* ^2^_adj_ (Model 1)=0.43, *R* ^2^_adj_ (Model 2)=0.39.

Asterisks indicate statistical significance:

* *p*<0.05

** *p*<0.01

*** *p*<0.001

a Ex-combatants were used as the reference group.

**Fig. 1:**
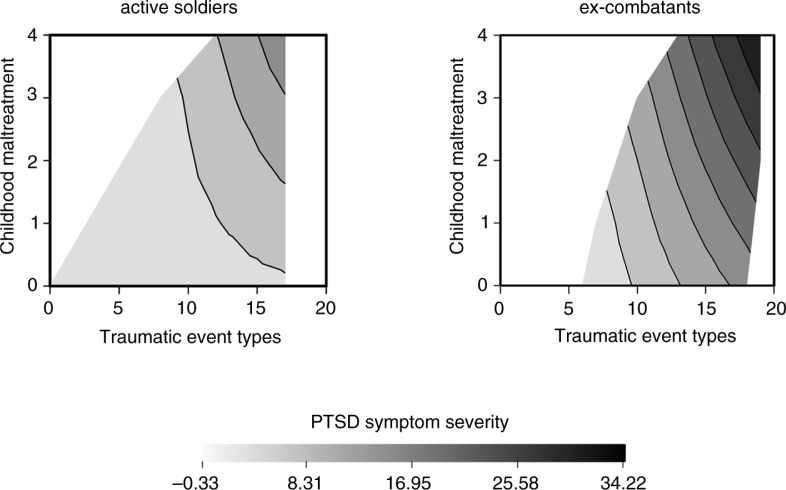
Relationship between the number of traumatic event types, childhood maltreatment, and PTSD symptom severity. At any given level of exposure to traumatic events, PTSD symptoms increase alongside childhood maltreatment and vice versa—with increasingly different types of childhood maltreatment, PTSD symptoms increase alongside the experience of multiple traumatic stressors. PTSD symptom severity is more pronounced in ex-combatants at all levels of both predictors.

Apart from the impact of exposure to *traumatic event types*, regression analysis also highlighted the significance of *childhood maltreatment*. The development of PTSD in relation to both classes of stressors is illustrated in Fig. 1, which shows the fitted PSS-I sum score based on the selected linear regression model (Model 1). The grey scale indicates the predicted *PTSD symptom severity*. Active soldiers and ex-combatants are separately plotted to enable comparison between the two groups. Both *childhood maltreatment* and *traumatic event types* cumulatively contribute to the development of *PTSD symptom severity. PTSD symptom severity* is more pronounced in ex-combatants at all levels of predictors.

### Predictors of appetitive aggression

Results of the multiple linear regression analysis for the prediction of *appetitive aggression* are presented in Table 2 (Model 2). The selected model revealed a main effect for the group variable. *Appetitive aggression* was higher in active soldiers than in ex-combatants. Furthermore, main effects were found for *traumatic event types* and *self-committed violence*. *Childhood maltreatment* was not an additional predictor. The final model included the significant interactions of childhood maltreatment *self-committed violence, group * *traumatic event types* and *group* * *self-committed violence*. The model accounted for 39% of the variance of *appetitive aggression* (*F* (7, 944) = 76.03, *p*<0.001, *f* ^2^=0.64). The maximum Variance Inflammation Factor did not exceed 2.11. Hence, multicollinearity could be neglected.

**Fig. 2:**
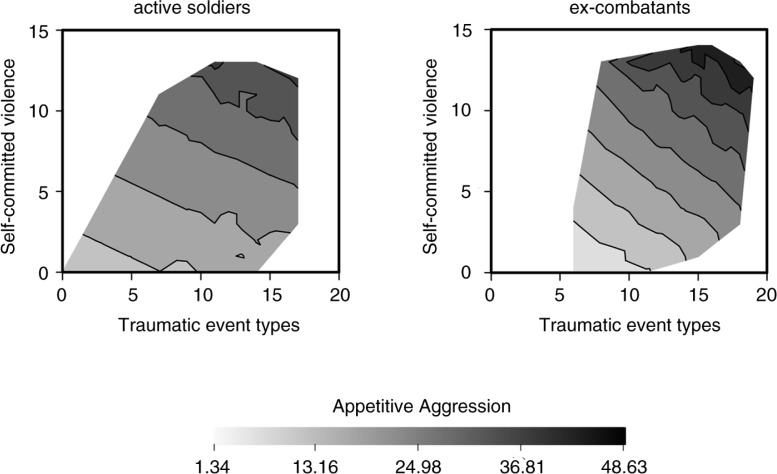
Relationship between the number of traumatic event types, self-committed violence, and appetitive aggression. With increasingly different types of traumatic events, appetitive aggression increases alongside self-committed violence. The highest levels of appetitive aggression are found in ex-combatants; however, at the same level of predictors, appetitive aggression is more pronounced in active soldiers.

Figure 2 illustrates the emergence of *appetitive aggression* in relation to *traumatic event types* and *self-committed violence*, separately plotted for ex-combatants and active soldiers. It shows the fitted AAS sum score based on the selected linear regression model (Model 2). The grey scale indicates the predicted values of *appetitive aggression*. Both predictors cumulatively contribute to the emergence of *appetitive aggression*. At the same level of predictors, *appetitive aggression* is more pronounced in active soldiers.

## Discussion

In this study, ex-combatants reported a higher exposure to different forms of traumatic events and childhood maltreatment than active soldiers. Furthermore, they specified having committed more violence. They were also more affected by PTSD symptoms and showed an overall higher degree of appetitive aggression.

Concordant with previous research, the “building block effect”—that is, the increasing likelihood of PTSD with an increasing variety of traumatic stressors—was found for both the ex-combatants and the active soldiers (Neuner et al., 2004). Even when controlling for the level of traumatic experiences and the exposure to childhood maltreatment, ex-combatants presented with more severe PTSD symptoms than currently active soldiers. Hence, active soldiers represented a more resilient group compared to ex-combatants. Even though we do not know the mental health status of ex-combatants and active soldiers directly after the end of the war, it is likely that this played a crucial role in their demobilization. Soldiers suffering from PTSD symptoms might have preferred to leave armed groups, while healthier individuals might have been interested in remaining in or being integrated into the army.

In accordance with previous research, childhood maltreatment was found to be an additional predictor of PTSD symptoms (Berntsen et al., 2012; Van Voorhees et al., 2012). The results indicated a moderating effect of childhood maltreatment: both kinds of adversities potentiated the risk of trauma symptoms, which is in line with other research in conflict regions where the interplay of different types of stressors such as family violence and war events was shown to enhance vulnerability to PTSD (Catani et al., 2010). However, childhood maltreatment and other traumatic events did not occur completely independently of each other: the minimum number of different traumatic events experienced increased with the extent of childhood maltreatment—that is, no participants reported childhood maltreatment along with no or only a few traumatic experiences. This result is in line with research emphasizing that maltreated children are at a higher risk of experiencing traumatic events outside the family, especially in combat situations (Iversen et al., 2007; King et al., 1996). Maltreated children probably leave families earlier to join armed groups. On the other hand, childhood maltreatment is also more common in families that are affected by war (Catani et al., 2008).

In contrast to other research (Bayer et al., 2007; Van Winkle & Safer, 2011), self-committed violence was not a predictor of PTSD symptoms in our study. Although exercising violent acts such as killing can be traumatizing, this is not necessarily the case for everyone or in every situation. Hecker and colleagues (2013) demonstrated that perpetrating violence is not traumatizing for combatants who had voluntarily joined an armed force. In our sample, this was the case for over 95% of the participants. Whether self-perpetrated violence is traumatizing for a person or not apparently depends on the particular situations and circumstances. Future research therefore needs to separately assess different types of violence (reactive vs. appetitive) and consider the range of emotions that was present when the violence was enacted.

In line with previous studies in post-conflict regions (e.g., Hecker et al., 2013), this study also demonstrated that high levels of appetitive aggression can generally be found in a combatant population. Self-committed violence was a strong predictor of appetitive aggression, which is consistent with prior studies demonstrating that committing violent acts fosters the perpetrator's perception of aggression as appetitive (e.g., Crombach & Elbert, 2014; Hecker et al., 2012). Traumatic events also predicted appetitive aggression. This effect is consistent with research that has emphasized an increase in aggressive behavior in adverse environments as an adaptive mechanism (e.g., Weierstall et al., 2011). Furthermore, the results of our study support the hypothesis that growing up in a violent environment is related to subsequent violent behavior. Even though childhood maltreatment itself did not directly predict appetitive aggression in our study, it moderated the effect of self-committed violence on the perception of aggression. Experiencing childhood maltreatment increased the association between self-committed violence and appetitive aggression. A possible explanation for this is that childhood maltreatment happens particularly in environments that favor violent behavior, which in turn socializes the mistreated children (Schwartz, Dodge, Pettit, & Bates, 1997).

At an absolute level, ex-combatants reported more appetitive aggression than active soldiers. However, when controlling for exposure to traumatic events and childhood maltreatment as well as for self-committed violence, the active soldiers showed a relatively higher degree of appetitive aggression. Again, this may have been due to selection effects during the demobilization process after the war. Individuals with higher appetitive aggression and at the same time lower PTSD symptom severity might be more likely to have remained in the army.

A methodological limitation of this study is the correlational design. It is thus not possible to prove causal inferences about the relations between variables. We also do not have a longitudinal picture of the onset and history of either PTSD or appetitive aggression. It could be, for example, that the PTSD was much worse in the immediate aftermath of the war, and has subsequently improved. Furthermore, for traumatic events and self-committed violence, we did not measure exactly when they occurred in the participants’ lives. As our data are based on subjective reports, there might be a potential bias due to retrospective recall or social desirability. We cannot rule out that active soldiers systematically under-reported symptoms of PTSD. This might have enlarged the observed difference between groups of active soldiers and former combatants. However, conducting interviews allowed us to get also a clinical impression, which supported the huge difference between the two groups.

In conclusion, the results of the present study indicate that childhood maltreatment is a severe risk factor that exacerbates the psychological consequences of violent conflicts in post-conflict or crisis regions. Self-committed violence frequently may not promote trauma-related disorders but poses a risk factor for the development of appetitive aggression. The level of appetitive aggression seems to be a source of potential risk in both former and active combatants. The present research disentangled and highlighted several important aspects of the complex interplay between different factors leading to PTSD and appetitive aggression.
